# The black sheep effect: The case of the deviant ingroup robot

**DOI:** 10.1371/journal.pone.0222975

**Published:** 2019-10-16

**Authors:** Andrew Steain, Christopher John Stanton, Catherine J. Stevens

**Affiliations:** MARCS Institute, Western Sydney University, Sydney, Australia; University of Milan, ITALY

## Abstract

The black sheep effect (BSE) describes the evaluative upgrading of norm-compliant group members (ingroup bias), and evaluative downgrading of deviant (norm-violating) group members, relative to similar outgroup members. While the BSE has been demonstrated extensively in human groups, it has yet to be shown in groups containing robots. This study investigated whether a BSE towards a ‘deviant’ robot (one low on warmth and competence) could be demonstrated. Participants performed a visual tracking task in a team with two humanoid NAO robots, with one robot being an ingroup member and the other an outgroup member. The robots offered advice to the participants which could be accepted or rejected, proving a measure of trust. Both robots were also evaluated using questionnaires, proxemics, and forced preference choices. Experiment 1 (N = 18) manipulated robot grouping to test our group manipulation generated ingroup bias (a necessary precursor to the BSE) which was supported. Experiment 2 (N = 72) manipulated the grouping, warmth and competence of both robots, predicting a BSE towards deviant ingroup robots, which was supported. Results indicated that a disagreeable ingroup robot is viewed less favourably than a disagreeable outgroup robot. Furthermore, when interacting with two independent robots, a “majority rule” effect can occur in which each robot’s opinion is treated as independent vote, with participants significantly more likely to trust two unanimously disagreeing robots. No effect of warmth was found. The impact of these findings for human-robot team composition are discussed.

## 1 Introduction

The ability of humans to work effectively in groups is a fundamental aspect of human life, allowing for civilised and productive society, while selectively bestowing survival advantages upon stronger and more cohesive collectives. With advances in robotics, traditional human working groups are becoming increasingly interspersed with artificial agents, in fields such as healthcare, the military, and transportation. Therefore, factors which may increase trust and rapport towards technological teammates are of increasing importance, as they may influence both the working relationship between man and machine, and the critical decision to rely on, or cease using a technological agent [1]. Furthermore, as robots shift from being automated tools to autonomous teammates, this raises questions concerning how co-workers respond to robots who offer viewpoints and advice that deviates from group standards, and thus the impact this may have on team performance.

### 1.1 Ingroup bias

Ingroup formation and dynamics is built upon similarities and biases. For example, managers are more likely to hire employees that are like themselves [2], an effect known as similarity or affinity bias. These tendencies to positively evaluate ourselves and fellow group members are the crux of ingroup bias, in which people generally favour and prioritise members of their own group (the ingroup), rating them more capable, friendly, and altruistic than corresponding members of another group (the outgroup) [3]. Thus, ingroup favouritism is considered a factor in issues ranging from prejudice and racism, to social and economic disadvantage of minority groups [4].

Ingroup bias is strongest on attributes most important to the ingroup [5], [6]. For example, Marques and Paez [7] found that military cadets showed a much stronger ingroup bias towards fellow cadets who conformed to codes of conduct identified as personally salient (e.g. loyalty, toughness), compared to those conformant with codes considered irrelevant (e.g. punctuality, neatness). Similarly, Marques, Yzerbyt, and Leyens [8] found Belgian students rated fellow ingroup students more favourably versus outgroup (Moroccan) students when they conformed to a behaviour valued by the ingroup specifically (attending university parties) compared to when they conformed to a behaviour valued by both groups (lending course notes).

While ingroup bias has been demonstrated using these real-world salient groupings [9], it has also been demonstrated in experimental settings using trivial and arbitrary differences such as shirt colour [10], the shape of a token [11], or ratings of paintings [12]. Ingroup bias has also been demonstrated in human-robot groups. Group membership has been manipulated via colour, with participants more willing to interact with an ingroup robot [13]. The nationality of a robot’s programmers can generate ingroup bias, with participants being more cooperative with a robot of the same nationality [14]. Ingroup bias can even be generated by influencing participants’ perceptions of their suitability for working in a human-robot team, with outgroup participants positioning themselves further away from a robot than ingroup participants [15].

Explicit measures of ingroup bias commonly involve ascription of group traits to assess intergroup stereotypes, and disparities in behaviour between ingroup and outgroup targets to measure discrimination [16]. Alternatively, implicit measures consider judgments and attitudes unconsciously activated by ingroup or outgroup targets. A form of implicit measurement used to assess attitudes is unobtrusive proxemics [17]. Proxemics measures focus on the awareness, use and organisation of space, and assess how one’s use of space (intentional or incidental) affect and indicate relationships with others [18]. Proxemics measures assert that greater immediacy (proximity) to others corresponds with more positive evaluations of them, with people maintaining closer distances to liked others, and further distances from those they dislike [19]. Research on prejudice has supported these assertions [4]. Word, Zanna, and Cooper [20] showed that White participants maintained greater distances from Black confederates as opposed to White confederates, and Bessenoff and Sherman [21] revealed that attitudes of thin participants regarding obese individuals were negatively correlated with their seating distance from an overweight experimental partner. In a human-robot interaction study, participants positioning themselves physically closer to an ingroup robot [15]. Proxemics thus allows for implicit measurement of attitudinal differences and preferences towards ingroups and outgroups, with people maintaining closer interpersonal distances to members of their ingroup versus members of an outgroup.

### 1.2 The black sheep effect

Where ingroup bias considers how ingroup members evaluatively upgrade fellow members who boost collective social identity, the black sheep effect (BSE) [8] investigates the evaluative ramifications for members who threaten group identity [22]. The BSE hypothesis asserts ingroup members should elicit more intense and polarising judgments than outgroup members, and hence the BSE posits that deviant unlikeable ingroup members will be derogated more than a respective similar outgroup member [23]. This polarisation of judgment towards ingroup members has been considered a form of ingroup favouritism whereby derogating negative members allows a collective to maintain group positivity and cohesion. Social identity theorists have consequently suggested that BSE’s and ingroup biases indicate the same core intention: maintenance of positive social identity [24].

The BSE has been repeatedly demonstrated in experimental conditions. For example, Travaglino, Abrams, de Moura, Marques, & Pinto [25] measured group reaction to defection of a team member to a rival team, with ingroup defectors rated significantly more negatively than their outgroup counterparts. Mendoza, Lane, & Amodio [26] found that ingroup members would be punished more harshly than outgroup members for violated fairness norms with respect to monetary bargaining. In a social drinking scenario, Lo Monaco, Piermatteo, Guimelli, & Ernst-Vintila [27] found that ingroup members who drank alcohol alone were more negatively evaluated than corresponding outgroup members.

While the BSE focuses on the potential harms, and responses to ingroup deviance, ingroup deviance and dissent does not necessitate negative group outcomes, and can even be beneficial for group decisions. Research on minority influence has suggested dissent can promote ingroup creativity, protect against complacency, and result in more measured collective thinking [28],[29]. Moreover, research suggests groups that include devil’s advocates make superior judgments [30], are more critical of information [31], and are insulated from making decisional errors [32]. Thus, the study of ingroup deviancy in human-robot working groups may hold important implications for humans working alongside robots in terms of both productivity and team cohesion.

### 1.3 Warmth and competence

Warmth and competence are dimensions on which both individuals and groups are assessed, with the Stereotype Content Model (SCM) proposing that group stereotypes are formed from these two dimensions [33]. Warmth considers qualities related to personal motive, including openness, goodwill, and trustworthiness, while competence includes qualities such as aptitude, ingenuity and talent [34]. The SCM proposes that people are predisposed to first assess a persons’ intent to either harm or help them (warmth), and to then secondly to judge the persons’ capacity to act on that perceived intention (competence). Warmth and competence have been widely researched, with these dimensions converging across survey, cultural, laboratory, and biobehavioral approaches [35]. Importantly for this study, warmth and competence have previously been manipulated to elicit BSE’s [36],[37].

#### 1.3.1 Warmth

Various nonverbal indicators of warmth have been identified throughout the social psychological literature. For example, ‘affiliative’ nonverbal behaviours such as head nodding, hand gestures, eye contact and forwards-leaning posture [38],[39]. Eye gaze is a particularly powerful determinant of liking between people when first introduced [40], with moderate levels of eye gaze favoured over consistent or no eye gaze altogether [41]. Moderately open-arm configurations are evaluated as warmer and more receiving, whereas closed arm configurations are cold, refusing, and unreceptive [42]. Warmth evaluations influence our decisions to approach or avoid others, a primary aspect of the social judgment process [43].

Robot gaze has been shown to have impact upon human behaviour. For example, robot gaze can influence the distance that people maintain towards robots [44]. Bergmann, Eyssel & Komp [45], found that virtual agents who gestured while interacting with participants were rated higher on warmth indices than non-gesturing avatars.

Demeure, Niewiadomski, & Pelachaud [46] showed that robots who display socially appropriate emotions were rated more believable, warm, and competent versus those which were socially inappropriate, or lacked emotional expression. Peters, Broekens, & Neerincx [47] developed a model of nonverbal behaviour to express both competence, and high and low warmth that was implemented on a Nao robot, with high warmth robots facing their audience with head high, and low warmth robots facing away with head low.

#### 1.3.2 Competence

Among people, competence includes qualities such as aptitude, ingenuity and talent. Competence evaluations generally follow warmth evaluations, due to an evolutionary need to assess another’s motive before their ability [34]. Whereas people are finely attuned to information that might discredit warmth judgements, such as manipulation or lying, competence judgments are far more responsive to positive evidence, as it is inherently more difficult to “fake” competence [48].

In human-robot interaction, the competence (i.e. performance consistency) of a robot, as measured by factors such as false alarm, reliability and failure rate, is the most valuable predictor of trust growth and maintenance, outweighing other attribute factors such as appearance and robot personality [49]. Furthermore, users more likely to intervene and manually control a low competence robot [1]. Similarly, a meta-analysis investigating the factors influencing trust in human-machine teaming found that reliable robot behaviours with low error rates increased user confidence in the system [50].

Lee, Lau, & Hong [51] suggest warmth and competence are prioritised dependent on task and robot appearance. They propose that humanlike robots should be perceived primarily in terms of warmth due to their human similarity, and robot warmth prioritised when a task involves social behaviour and roles (e.g., sales assistant, cashier). Conversely, machine-like robots should be conceived primarily in terms of competence due to their mechanised appearance, with competence prioritised when the robot’s role is goal-oriented (e.g., soldier, security guard). Such differential treatment is due to the evocation of different mental models based around appearance and perceived capacity [52].

## 2 The present study

While research using technological devices has shown the presence of ingroup biases towards technological devices, research has yet to find a BSE towards a technological agent. The present study involved two experiments: Experiment 1 aimed to establish ingroup bias, a necessary precursor to the BSE, while Experiment 2 aimed to establish a BSE towards an ingroup robot that is deviant in both warmth and competence.

### 2.1 Hypotheses

In Experiment 1, participants interacted with two robots manipulated by grouping only. The aim of Experiment 1 was to establish that our manipulation of ingroup and outgroup membership generated an ingroup bias, a necessary precursor for a BSE in Experiment 2.

**H1**: **participants will show favouritism towards the ingroup robot**.

Experiment 2 manipulated each robots’ grouping, warmth and competence, with the aim of producing a BSE for an ingroup robot low in warmth and competence.

**H2**: **A deviant ingroup robot will produce a BSE**.

**H3**: **As research has shown that robot reliability is the most important factor determining confidence and trust in robots, competence will be more important than warmth in eliciting a BSE**.

## 3 Experiment 1: Intergroup bias

### 3.1 Cover story

A cover story was told to participants (1^st^ year undergraduate psychology students at Western Sydney University) to manipulate group membership. Upon arrival, participants were told the following cover story:

“*Today you’ll be playing the shell game with two robots to help you*. *The reason we’ve got two robots is that even though they appear identical*, *and they were programmed by the same person*, *they use two different algorithms from two different sources*. *This is ‘Eng bot’*, *and this is ‘Psyc bot’[experimenter points to robots]*. *‘Eng bot’ uses an algorithm designed by Engineers from the University of Cape Town in South Africa that is based around engineering principles for motion tracking*. *‘Psyc bot’ uses an algorithm designed by Cognitive Psychologists from the MARCS institute here at Western Sydney University*, *Bankstown campus*, *based around insights from psychology about how the vision system works*.”

In truth, both robots were teleoperated in a Wizard-of-Oz setup, of which the participants were unaware. Banners displaying the university and discipline of each robot were placed in front of each robot for participant recognition. Furthermore, participants were told that after the shell game, there would be another short task (however, in truth, there were no remaining task, and the pretence of this second task was to gain a proxemic measure, described in more detail in Section 3.6.3). Participants were instructed to achieve the highest score possible, and to select the answer they believed most likely to be correct, whether it be their own answer, or a different answer provided by one of the robots.

### 3.2 Shell game task

**Fig 1:**
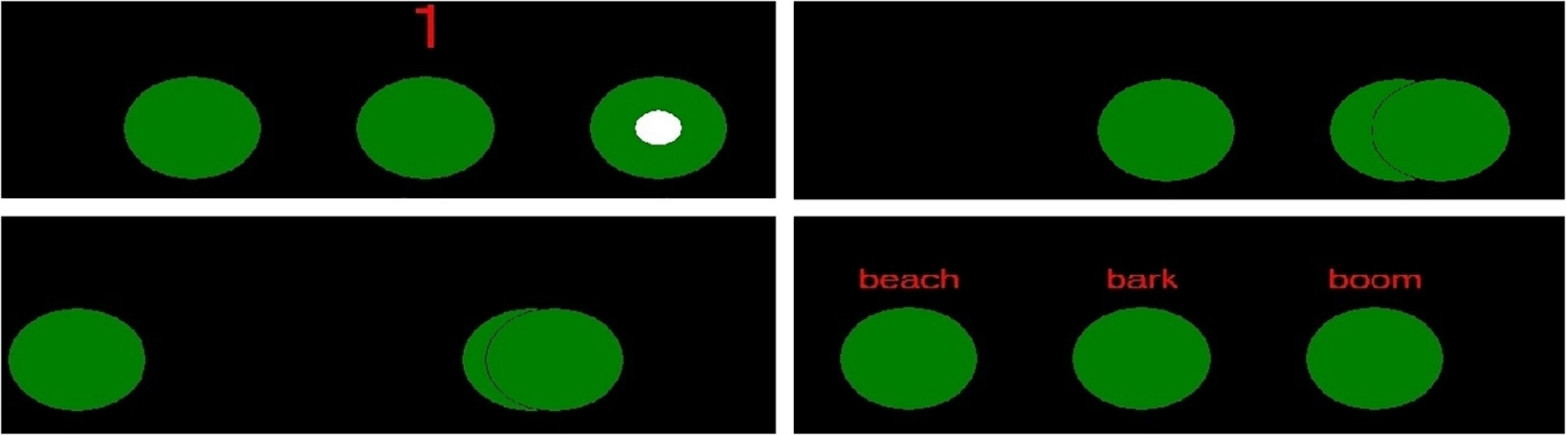
The shell game stimuli. Top Left: each trial begins with a “3-2-1” countdown. The white circle indicates the cup to be tracked. Top Right and Bottom Left: The cups move horizontally for 4 seconds, with changes of direction, overlap and occlusion, creating uncertainty as to the target cup’s true location. Bottom Right: When the cups have finished moving, participants and robots identify their answer by saying the word above the cup that they believe to be hiding the white circle.

Participants in both experiments played an interactive video game adaptation of the classic “shell game” (aka the “cup game”) in which an object is hidden under one of three cups, with those cups then rapidly shuffled to create ambiguity as to the object’s location (see Fig 1). Participants were told they would be playing the shell game in a team with the robots, and both robots would provide suggestions regarding the target location, which may match or disagree with the participant’s initial answer. The objective of the game was for the participant to get highest score possible, by choosing the answer on each trial that they believed to be most likely to be correct, albeit their own answer, or a suggestion provided by the robot(s).

**Fig 2:**
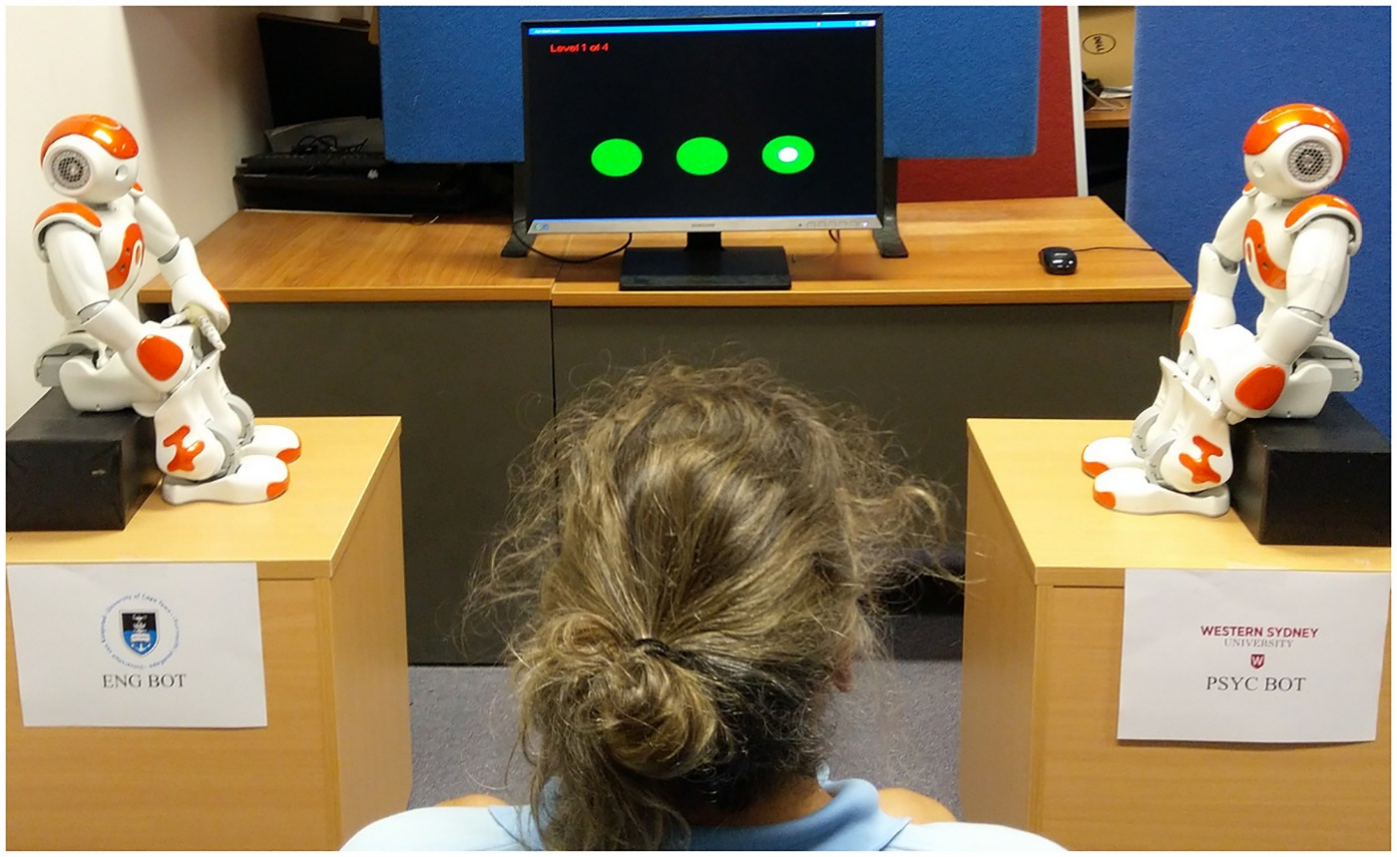
The shell game setup. Participants sit on a chair, facing the shell game stimuli with a robot to their left and right, with each robot clearly identified as either “Psyc Bot” or “Eng Bot”. Robot positions (left versus right) and speaking order were both counterbalanced. When each shell game trial begins, the robots would turn their heads towards the monitor to view the shell game. When speaking to a participant, the robot speaking would turn its head to face the participant.

Participants sat facing the shell game display with a robot on their left and right (see Fig 2). For each trial, the cup shuffling process took four seconds, after which a word appeared above each cup. After the cups stop moving, the robots would alternate turns in asking the participant “What is your answer?”, and participants would identify their answer to the robot using the word that appeared above the cup they believed to be hiding the object. The turn-taking order of the robots (i.e. which robot asked first) was counterbalanced, as was the physical location of the robots (i.e. ingroup robot on the participant’s left or right, and vice versa). The robot’s speech was produced using the Nao’s text-to-speech engine using the default shape and speed settings.

**Fig 3:**
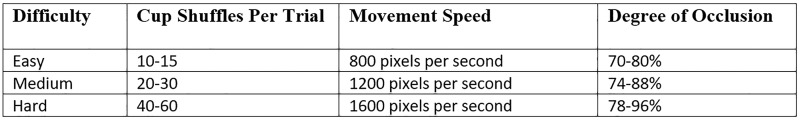
Task difficulty. Shell game difficulty was manipulated by speed of cup movement, the number of shuffles that occurred when two cups were overlapping, and the degree of occlusion when cups were shuffled (for example, a partial to almost full eclipse).

Game trials comprised three difficulty levels (Easy, Medium, and Hard), with difficulty determined by the speed of cup movement, the number of cup “shuffles” per trial (changes of direction in cup movement that occurred while cups are overlapping), and the degree to which the cups overlapped when being shuffled (see Fig 3). Trial Difficulty was not considered as an independent variable. Participants completed 40 trials in 4 blocks of 10 (2 easy, 2 medium, 6 hard per block). After each block of 10 trials, participants were given a one-minute break. To restart the game participants could either say “Eng Bot resume game” or “Pysc Bot resume game”, with their preference (ingroup or outgroup robot) being recorded.

On some trials, one or both robots would disagree with the participant, providing an alternative answer. If a robot disagreed with the participant’s answer the robot would say, “I disagree. I think it is <different answer>”. Lastly, after presenting differing answers, the robot whose turn it was to initiate dialog would ask “What is your final answer?”. Each participant’s rate of answer change to a robot’s suggested answer provided a measure of trust.

The robots were programmed to disagree with a participant’s answer in the following circumstances:

- On the 8 Easy trials, the robots would only disagree with the participant if the participant’s initial answer was incorrect (both robots would provide the correct answer). Note, Easy trials were not considered in data analysis. On most Easy trials, participants and robots would unanimously agree.
- On the 8 Medium trials, regardless of the participant’s initial answer, one robot would provide the correct answer and the other robot would provide an incorrect answer, with incorrect answers split evenly between the Ingroup and Outgroup robots
- On 16 of the 24 Hard trials, the robots disagreed with each other and the participant, meaning three different answers were provided. On 6 of the 24 Hard trials, the two robots provided identical answers that differed from the participant’s initial response. On the remaining 2 Hard trials, the two robots both agreed with the participant’s initial response.

### 3.3 Procedure

Participants were tested individually. On arrival, participants were told the cover story, and how to play with shell game with the robots as teammates. The experimenter then initiated a block of three practice trials by stating verbally “robots, begin practice trials”, whereby a confederate experimenter remotely initiated the game (to maintain the participant’s perception of the robots’ autonomy). Once the practice trials were completed, the experimenter left the room to allow the participant to complete 40 trials of the shell game alone with the robots. Between each block, participants were prompted with on-screen instructions to resume the game by saying either “Psyc Bot resume game” or “Eng Bot resume game”. Participants were given an on-screen score update after the second block of trials (i.e. after 20 trials), and a final score update at the completion of 40 trials.

During the shell game, an experimenter was in an adjacent room, hidden from the participants, controlling the robots via a Wizard-of-Oz setup. Participants’ initial and final responses for each trial were logged, as was their choice to choose either Psyc Bot or Eng Bot to resume the game after each block.

**Fig 4:**
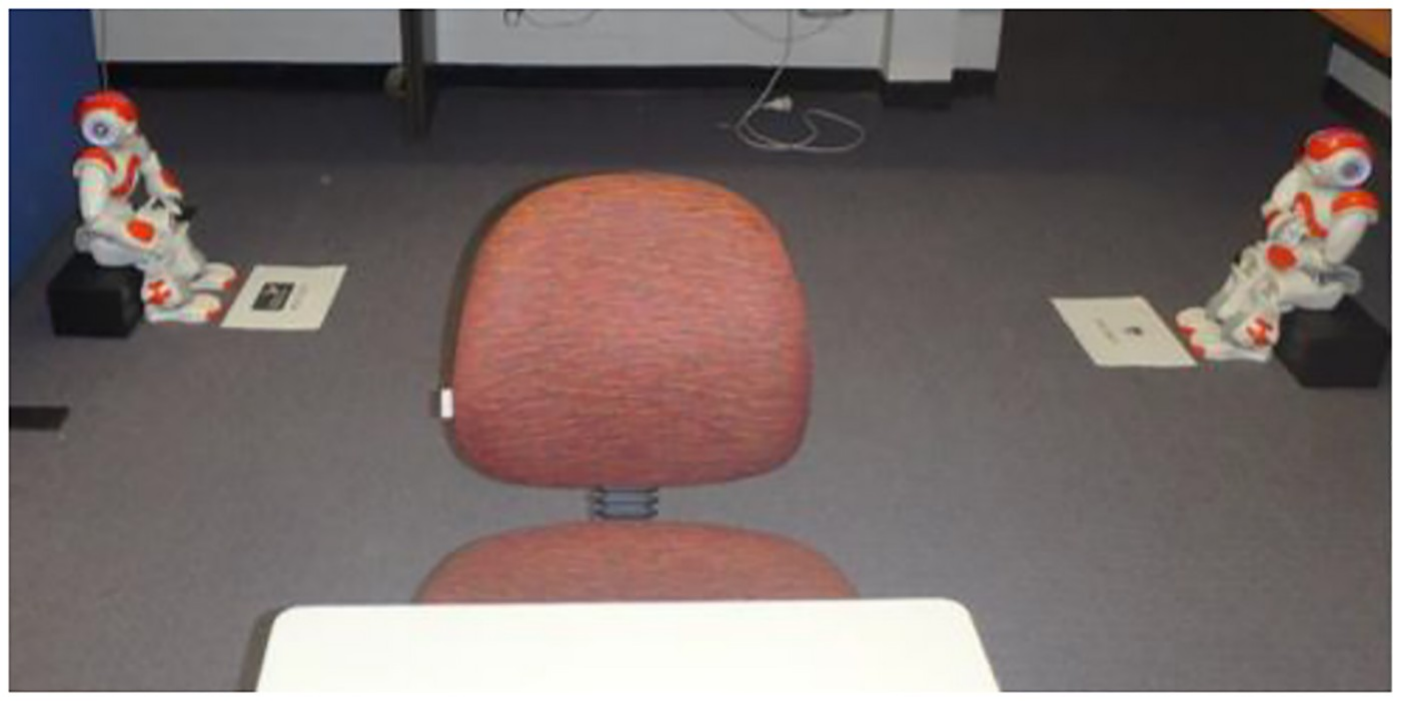
Laboratory setup of the two robots for the proxemics measure. The participant was asked to move a wheeled office chair towards the robots and take a seat “anywhere you feel comfortable”.

After completion of the shell game task, the experimenter re-entered the room. The experimenter then moved the two robots to the positions described in Fig 4. The participant was asked to move a wheeled office chair towards the robots and take a seat “anywhere you feel comfortable”. The experimenter then left the room under the pretence of “collecting some equipment”, and then after 30 seconds the experimenter would re-enter the room. Participants were then told that experimenter had “made a mistake”, and that they need to leave their chair where it is and move to Station 1 to complete a questionnaire. The location of the participant’s chair was used as a proxemic measure (the distance to both Psyc Bot and Eng Bot was measured).

Once participants had completed the questionnaire, they were instructed to choose one robot to interact with but were not told what the task would involve. After choosing a robot (the participant’s interaction choice was recorded), the participants were instructed to tap the bumper button on that robot’s foot, on which the robot provided a spoken message thanking the participant for their participation. The participant was then debriefed, marking the end of the experiment. The entire experiment took 25–30 minutes per participant.

### 3.4 Participants

The experiment was advertised on Western Sydney University’s “Research Participation System”, which provides a listing of experiments that first year psychology undergraduate students can participate in, and in return receive course credit. Participants were required to have normal vision (or corrected to normal vision, e.g. glasses), and the ability to speak English. Through the university’s research participation system, a total of 18 Western Sydney University first year psychology undergraduate students were recruited (4 males, 14 females, with a mean age of 22.3 years, SD = 7.37).

### 3.5 Design

A one-way, within-subjects design was employed, with robot group the single independent variable (2 levels, ingroup and outgroup). Five dependent variables measured grouping preference: answer change to each robot’s suggested answer, robot preference for shell game resumption, robot interaction task preference, a proxemics measure, and questionnaire responses.

### 3.6 Measures

#### 3.6.1 Shell game: Trust

Trial responses were logged automatically via software. Participants initial responses were recorded, and their final responses were recorded on trials in which a robot(s) to disagreed with them. “Trust” was measured as the rate that participants changed their initial response to a robot’s response when a robot disagreed with them.

#### 3.6.2 Questionnaire

Participants completed the Godspeed indices [53], along with 8 additional items assessing vision and competence, and a four-item warmth scale [33]. Identical questions were completed for both Psyc Bot and Eng Bot. All items were answered via a 5-point Likert scale. Lastly, participants answered 3 hand-written questions which asked which robot they preferred and why, which robot they thought was better at the shell game, and what prior experience (if any) they have had with robots.

#### 3.6.3 Proxemics

Robots were positioned atop of rectangular boxes (i.e. “chairs”) on the floor, 2.2 metres apart, facing toward each other, perpendicular to the original location of the chair (see Fig 4). Participant distance from each robot was measured to the centre of the chair.

#### 3.6.4 Resume game preference choices

Participants’ verbal selection of either Eng Bot or Psyc Bot to restart the Shell Game after each block of trials (3 times per participant).

#### 3.6.5 Interaction preference choice

The choice of robot (Eng Bot or Psyc Bot) when prompted by the experimenter to choose a robot to interact with, after completion of the Shell Game task, under the pretense of completing a second remaining task (the nature of which was yet to be described to the participant).

### 3.7 Results: Experiment 1

#### 3.7.1 Shell game

A total of 720 trials were completed (18 participants, 40 trials per participant). Participant responses were pooled and analysed to derive mean participant trust rates for both robots (the rate participants changed their initial answer towards a robot answer), mean unanimous trust rates (response change when both robots provided an identical answer different to the participant), and mean disagreement trust rates (response change when robots both offered a different suggestion to participants).

Hypothesis 1 predicted participants would more frequently select ingroup robot answers compared to outgroup robot answers. A series of one-way repeated measures analyses of variance (ANOVA) were performed on shell game data to determine mean differences between Psyc Bot and Eng Bot persuasion rates. There was no significant difference between individual robot trust rates *F*(1, 17) = .168, *p* = .687, *n*_*p*2_ = .01, thus the first hypothesis was not supported.

An unexpected result arose from the shell game. By having both Psyc Bot and Eng Bot provide suggested answers on each trial, this resulted in three possible disagreement scenarios: a) both robots would provide different disagreeing answers; b) one robot would disagree with the participant, and the other agreed with the participant; c) both robots would provide the same answer in disagreement with the participant. In the latter two scenarios, a 2 vs 1 group dynamic occurs, with two of three players providing the same answer in disagreement with the remaining player (albeit human or robot). In the circumstances, an unintended “majority rules” effect occurred, with a significant difference between individual robot trust rates and unanimous trust rates *F*(1, 17) = 22.05, *p* < .001, *n*_*p*2_ = .57, with participants more likely to change their initial answer when robot answers were unanimous (*M* = .54, *SD* = .34) than to either robot individually (*M* = .22, *SD* = .24). Secondly, a significant difference was found between unanimous and divided disagreement trust rates *F*(1, 17) = 43.65, *p* < .001, *n*_*p*2_ = .72, with participants significantly more likely to change their decision to a robot’s answer when both robots provided identical answers (*M* = .54, *SD* = .34) versus when robot answers differed (*M* = .00, *SD* = .00). For both relationships, the observed power was above the 0.7 recommended by Hills (2011) (1.0 for both unanimous versus individual trust & unanimous versus divided trust analyses).

#### 3.7.2 Proxemics

H1 expected participants to maintain closer distances towards the ingroup robot versus the outgroup robot. A paired samples *t*-test was conducted on 18 participants to assess whether participants maintained closer distances to the ingroup robot over the outgroup robot. The *t*-test revealed a significant difference between participant distance towards robots *t*(17) = -2.06, *p* = .028. Participants maintained a closer distance towards Psyc bot (*M* = 135.83cm, *SD* = 22.41) compared to Eng bot (*M* = 150.00cm, *SD* = 26.40), providing support for Hypothesis 1.

#### 3.7.3 Questionnaire

H1 predicted participants would rate the ingroup robot more favourably across questionnaire items. To analyse questionnaire responses, combined means were first derived from the eight items of interest (i.e., animacy, anthropomorphism, likeability, intelligence, safety, trust, vision and warmth). A series of paired-samples *t*-tests were then run on these item means for the responses of 18 participants to assess preference for the ingroup robot. The *t*-tests revealed participants rated Psyc bot (*M* = 4.12, *SD* = .56) as significantly more intelligent than Eng bot (*M* = 4.04, *SD* = .73); *t*(18) = 1.96, *p* = .034. No significant differences were found between mean ratings of robots on the remaining factors, indicating Hypothesis 1 was partially supported.

#### 3.7.4 Resume game preference choices

H1 anticipated that participants would more frequently select the ingroup robot to resume the shell game. A one-way chi-square goodness-of-fit test was conducted on data to assess differences in frequency of Psyc bot versus Eng bot selection at resume game intervals. Data from three participants was discarded due to non-understanding of experimental instructions (participants said “Psyc-Eng bot resume game” rather than specifying a single robot). Using a .05 alpha, the chi-square indicated a significant difference between robot selection χ2 (1, *N* = 45) = 9.80, *p* = .002, with Psyc bot (33, 73.33%) chosen more often compared to Eng bot (12, 26.67%) to resume game during intervals, in support of H1.

#### 3.7.5 Interaction preference choice

H1 anticipated that participants would more frequently select the ingroup robot to interact with on the blind interaction task. A one-way chi-square goodness-of-fit test was also performed on data from 18 participants to assess differences in frequency of Psyc bot versus Eng bot selection for the interaction task. Using a .05 alpha, the chi-square indicated a significant difference between robot selection, χ2 (1, *N* = 18) = 6.37, *p* = .012, with Psyc bot (15, 83.33%) being chosen more often by participants compared to Eng bot (3,16.67%), supporting H1.

### 3.8 Experiment 1: Conclusion

The aim of Experiment 1 was to demonstrate ingroup bias, a necessary precursor for a BSE. Against expectation, there was no difference found in participant trust towards the ingroup versus the outgroup robot, an instead a majority rules effect occurred. However, participants rated the ingroup robot more favourably on questionnaire items, maintained closer interpersonal distances to the ingroup robot, and more frequently selected the ingroup robot when presented with a binary preference choice between Psyc Bot and Eng Bot, thus demonstrating the cover story and procedure was capable of inducing ingroup bias among participants.

## 4 Experiment 2: The black sheep effect

The aim of Experiment 2 was to induce a black sheep effect, by having participants play the shell game with a deviant robot (i.e. a robot low in warmth and competence). Furthermore, a secondary aim was to determine whether warmth or competence has more impact with respect to deviance in a humanoid robot.

Experiment 2 was identical to Experiment 1 with respect to the cover story, the shell game task, and dependent variables. However, Experiment 2 differed to Experiment 1 in the following ways:

1. To avoid a majority rules effect during the shell game task, participants only interacted with one robot per trial, with the two robots (ingroup and outgroup) taking alternating turns to interact with the participant (with start order counterbalanced).
2. Independent variables related to warmth (high/low) and competence (high/low) were introduced to robot behaviour.

### 4.1 Cover story

The same cover story as used in Experiment 1 (described in Section 3.1), was used in Experiment 2.

### 4.2 Shell game task

The shell game task was identical to Experiment 1 (described in Section 3.2), with the exception that to avoid the unintended majority rules effect that occurred in Experiment 1, participants interacted with only one robot per trial, with the robots taking alternating turns to interact with the participant over the 40 trials. During the shell game, one robot asked the participant for their answer, then provided an answer of its own, before initiating the next trial. This procedure was then repeated by the other robot, across the series of 40 trials. This process was counterbalanced across trials, meaning participants interacted with both robots for 20 game trials each.

### 4.3 Procedure

The procedure was identical to Experiment 1, except for changes to the shell game task described in Section 4.2, and changes to the independent variables and experiment design (described in Sections 4.4).

### 4.4 Design

A 2 x (2 x 2) mixed factorial within-between design was utilised, with robot grouping the single within-subjects factor, (ingroup and outgroup). Competence (high and low) and warmth (high and low) comprised the two between-subjects independent variables.

#### 4.4.1 Warmth

**Fig 5:**
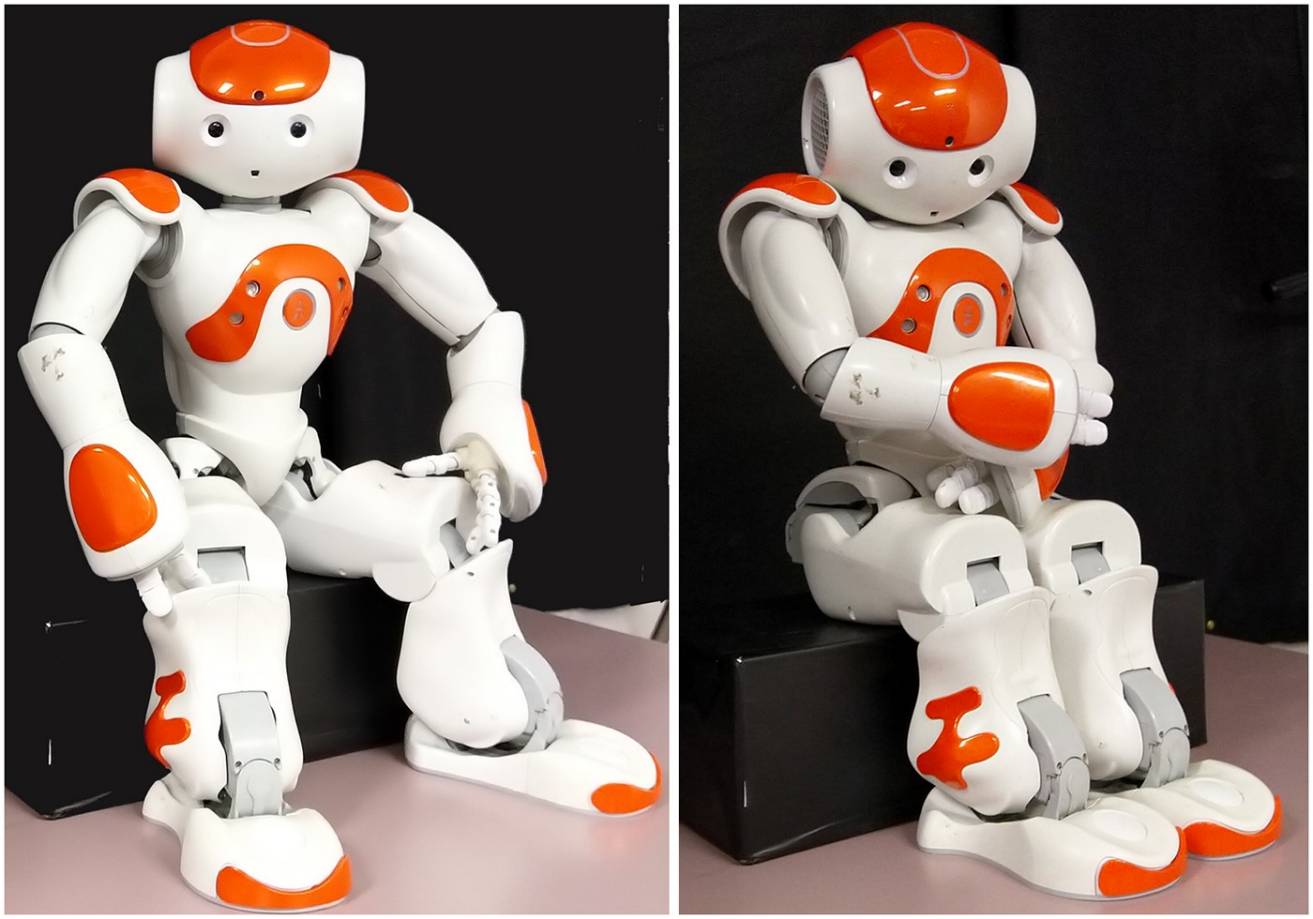
Nao robots with warm (left) versus cold (right) body positioning. Warm robots had an open stance and maintained direct gaze, while cold robots had crossed arms, closed legs and averted gaze.

Robot warmth was manipulated via robot head positioning, eye gaze, body leaning and limb positioning, as shown in Fig 5. High warmth robots had upright head positioning and direct eye gaze, leaned inwards and had open limb configurations. Low warmth robots had downwards head positioning, averted eye gaze, leaned backwards and had crossed arms and closed legs.

#### 4.4.2 Competence

Robot Competence was manipulated via the rate of robot correct responses during trials, which in turn impacted how frequently the robot disagreed with the participant, with low competence robots disagreeing more with participants than high competence robots. High-competence robots answered 36 from 40 trials correctly (90% accuracy, 1 incorrect hard response per block of 10 trials). Low-competence robots conversely answered only 28 from 40 trials correctly (70% accuracy), and incorrect answers were distributed across trial difficulty (3 hard mistakes on the first and third block; 2 hard, 1 medium mistakes on the second and fourth block of trials). Accuracy was manipulated in this way to create realistically competent and incompetent behaviours that were neither infallible nor completely erroneous.

### 4.5 Participants

Participants comprised 72 first year psychology students from the University of Western Sydney (18 male, 62 female, *M*age = 22.44 years, *SD* = 6.04). Sourcing and selection requirements were the same as in Experiment 1, with participants receiving course credit for their participation.

**Fig 6:**
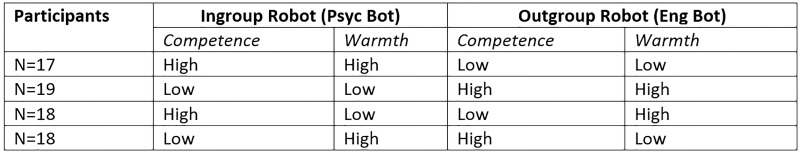
Participant group allocation for Experiment 2.

Participants were allocated to one of four conditions (counterbalanced) based around ingroup and outgroup robot warmth and competence (see Fig 6): high warmth and competence ingroup, low warmth and competence outgroup (ingroup bias condition); low warmth and competence ingroup, high warmth and competence outgroup (BSE condition), and two mixed conditions: low warmth and high competence ingroup, high warmth and low competence outgroup and high warmth and low competence ingroup, low warmth and high competence outgroup.

### 4.6 Results: Experiment 2

#### 4.6.1 Shell game: Trust

**Fig 7:**
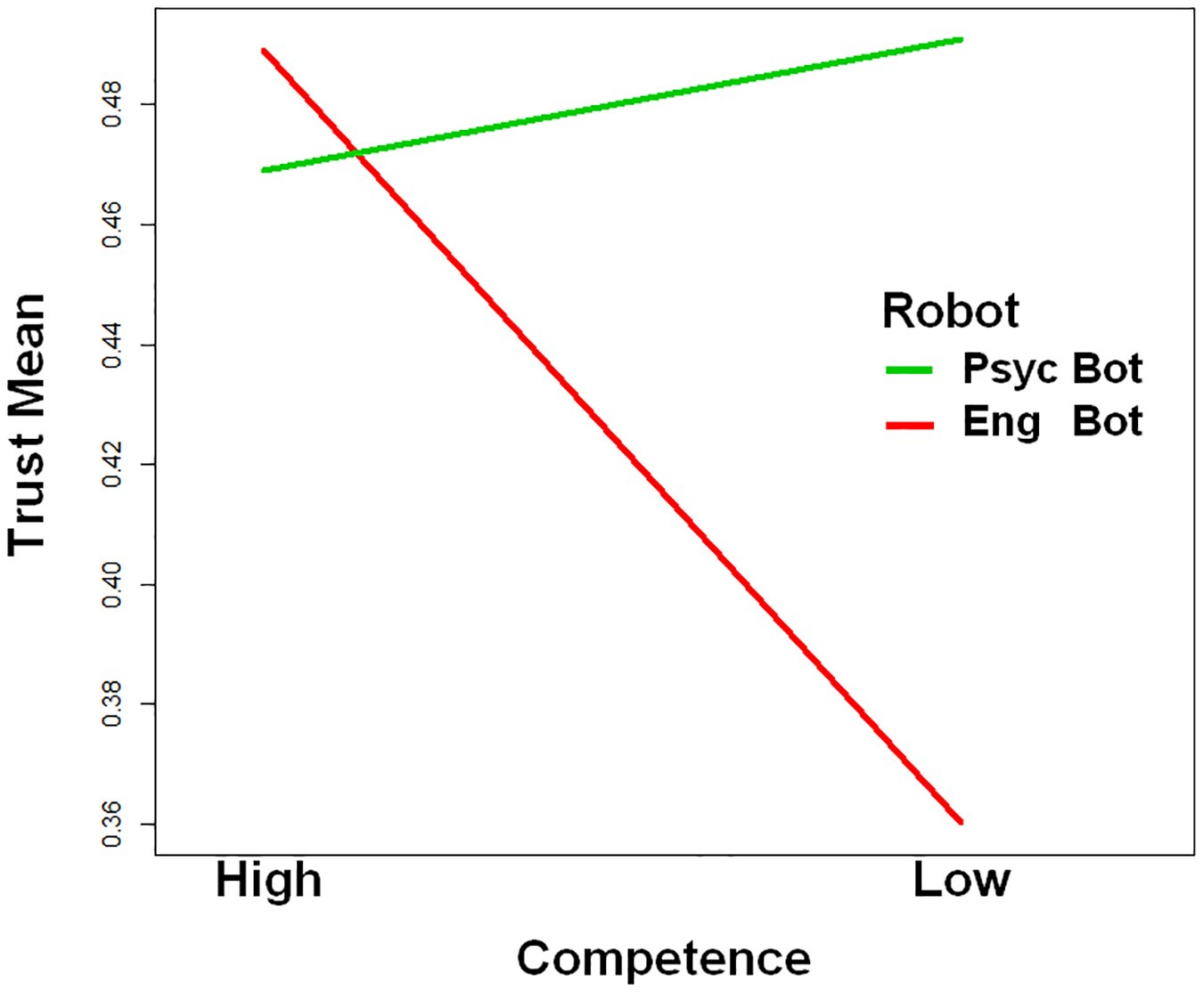
Interaction between Group and Competence for trust towards each robot during the shell game. Low competence only impacted trust for outgroup robots, with competence having no significant effect on ingroup robots.

A total of 2930 trials were completed (72 participants, 40 trials per participant). A mixed repeated measures ANOVA was conducted with robot trust (ingroup and outgroup) as the within-subjects factor, and warmth (high, low), competence (high, low) as between-subject factors. The ANOVA showed a significant interaction between group membership and competence, *F*(1,68) = 9.28, *p* = .003 *n*_*p*2_ = .12, as shown in Fig 7. Post-hoc analysis revealed that competence only had a significant effect upon outgroup robots, with low competence outgroups trusted less than high competent outgroup robots, *F*(1,68) = 4.95, *p* = .029 *n*_*p*2_ = .12. There was no significant difference in trust means between ingroup robots of different competences. No significant effects related to warmth were found *F*(1, 68) = .271, *p* = .605, *n*_*p*2_ = .00. H2 was not supported, and instead a negative bias towards outgroup low competence robots was demonstrated. For the group by competence relationship, the observed power (0.85) exceeded the 0.70 recommended by Hills (2011).

#### 4.6.2 Proxemics

Results showed a significant main effect of group membership on participant distance between robots, *F*(1, 68) = 6.34, *p* = .014, *n*_*p*2_ = .09, with participants on average positioning themselves closer to Eng Bot (*M*distance = 138.71cm, *SE* = 3.41, 95% CI [131.91, 145.50]) than Psyc Bot (*M*distance = 148.99cm *SE* = 3.54, 95% CI [141.94, 156.04]). A significant interaction was also found between group membership and competence, *F*(1, 68) = 4.29, *p* = .042, *n*_*p*2_ = .60, with participants positioning themselves significantly closer to the outgroup Eng Bot (*M*distance = 133.61cm, *SD* = 28.55) than the ingroup Psyc bot (*M*distance = 151.00cm, *SD* = .30.49) when Psyc Bot competence was low, thus supporting H2 and a BSE. No significant effects were found for warmth, thus supporting H3 that competence is more important than warmth in eliciting a BSE.

#### 4.6.3 Questionnaire

**Fig 8:**
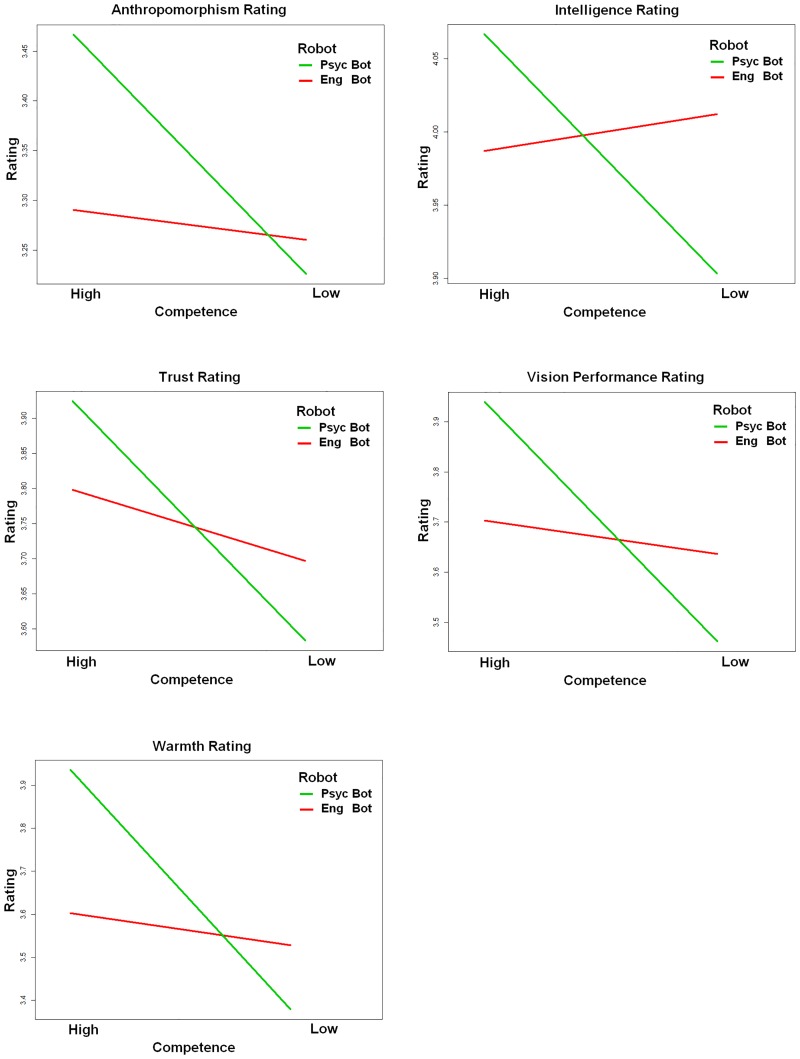
Significant interactions found via ratings measures for group and competence. Significant interactions were found for Group and Competence on ratings of robot anthropomorphism, intelligence, trust, vision performance, and warmth after the Shell Game. Low competence ingroup robots were rated more negatively than low competence outgroup robots, thus supporting a BSE.

A series of mixed repeated measures ANOVAs were run on item means for the responses of 72 participants for their rating of anthropomorphism, intelligence, likeability, safety, animacy, warmth, vision system performance, and competence. A significant main effect of anthropomorphism was found, *F*(1,72) = 5.34, *p* = .024 *n*_*p*2_ = .70, with participants rating Psyc bot (*M* = 3.38, *SD* = .83) as significantly more humanlike than Eng bot (*M* = 3.25, *SD* = .82). Furthermore, significant interactions between competence and group membership were found for anthropomorphism, *F*(1,71) = 9.54, *p* = .003 *n*_*p*2_ = .12, intelligence, *F*(1,71) = 6.37, *p* = .014 *n*_*p*2_ = .08, trustworthiness ratings, *F*(1,71) = 9.12, *p* = .004 *n*_*p*2_ = .11, warmth, *F*(1,71) = 12.01, *p* = .001 *n*_*p*2_ = .15, and vision performance, *F*(1,71) = 11.83, *p* = .002 *n*_*p*2_ = .14. As shown in Fig 8, these findings supported H2 and existence of a BSE, with Psyc Bot being rated more negatively than Eng Bot when competence was Low. No significant effects were found for warmth, or ratings of perceived safety or likeability. The lack of significant findings for warmth provides support H3, that competence is more important than warmth in eliciting a BSE.

#### 4.6.1 Resume game and interaction choice

Logistic regression analyses were conducted on both resume game and interaction choice data to assess for a BSE, and to explain the odds of participants selecting either Psyc bot or Eng bot based around the predictors of warmth and competence. In neither model were warmth or competence found to significantly increase the likelihood of robot selection, indicating hypotheses two and three were not supported.

### 4.7 Summary of results

A BSE was demonstrated with proxemics and a variety of questionnaire measures, but only with respect to an ingroup robot deviant in competence, with warmth having no impact. Participants more frequently trusted and favourably rated ingroup robots versus outgroup robots when they displayed high competence, but the opposite was true for low competence ingroup robots. Participants maintained further distances from ingroup robots low in competence compared to outgroup robots low in competence.

## 5 Discussion

The aim of Experiment 1 was to produce ingroup bias, a necessary precursor for a BSE. In Experiment 1, ingroup bias was demonstrated via proxemics and participant preferences for interaction with the ingroup robot. The bias towards Psyc Bot echoes previous findings in which robot grouping has elicited preferential behaviour [13],[14]. This supports the notion that social identity processes operate towards technological agents, as participants appeared motivated to upgrade fellow (robot) ingroup members to boost collective social identity and personal esteem.

An unintended “majority rules” effect occurred in Experiment 1 during the shell game task, with participants treating each robot as an independent voting entity, and thus tending to choose the answer which received the most votes. Conformity research posits that consensus denotes correctness [54] and provides the most direct means of goal achievement [55]. Hence when presented with a unanimous suggestion from two robots, participants appeared to submit to normative influence and align with the majority [56], rather than select an answer they objectively considered correct. Conversely, when robots provided different answers to participants, and constituted separate minorities, participants made their own judgment in the absence of a clearly endorsed response. This differs from the findings of Brandstetter et al. [57], who showed no effect of robot unanimity towards participant conformity in a line estimation task. Brandstetter et al. manipulated robot appearance and behaviour to individuate robots, theorising participants would perceive a group of heterogeneous robots as more convincing when their responses converged. Conversely, the present study differentiated robots by cover story only, representing the two robots as task-specialists differentiated by programming. This may have led participants to view the unanimity of two ostensible ‘experts’ as signifying correctness. This also suggests that the mental models of robot individuation and ability constructed by individuals may be more potent determinants of their decision to trust robots versus more superficial characteristics like appearance. As this is the first known study to find such a conformity effect using robots, future studies may benefit from further investigation into the impact of robot cover story upon user perceptions.

In Experiment Two, a BSE was demonstrated by participants physically distancing themselves further away from a low-competence ingroup robot than low-competence outgroup robot, and by more negatively rating a low-competence ingroup robot than a low-competence outgroup robot on perceptions of anthropomorphism, intelligence, vision system performance, and trust. This finding supports Hancock et al. [49] who found that robot performance is the most salient determinant of user satisfaction and reliance on technological agents. Whereas warmth is the primary dimension of social judgment in humans [34], our results suggest a robot’s competence was the key dimension of robot evaluation. As competence informs an agents’ ability to act upon their motives, this suggests the primary concern of those working alongside technological agents is the agents’ capacity to accomplish their goals. Considering technological agents are not yet commonplace in most professions, people may be understandably curious regarding the potential benefits and harms of working alongside technological teammates. For instance, though the NAO robots used in the current study were deliberately non-threatening in appearance and behaviour, in more applied settings (e.g., industrial contexts) robots can present a greater hazard for users due to the more powerful nature of such machinery [58].

An important caveat for these findings must be noted. As participants never receive feedback regarding which shell game trials they or the robot answered correctly or incorrectly, participants are unable to objectively assess the competence of themselves or the robots. As a low competence robot would disagree with participants more than a high competence robot, low robot competence may have been perceived as frequent robot dissent, rather than the robot’s performance in correctly identifying the target object. However, ingroup member dissent can nevertheless threaten ingroup social identity [59], and thus be used as a basis for the BSE [60]. Moreover, the competence of others is often based on perceptions and stereotypes of their ability rather than objective performance [48]. As the factors underlying people’s judgment of robots remain ambiguous [51], investigation into robot dissent may present an avenue towards elucidating the norms of human-robot interaction.

A BSE based on robot dissent supports the idea that depersonalised social identity operates in human-robot working groups. Ingroup members expect fellow members to conform with group norms and reciprocate the behaviour shown towards them [61]. Thus, when an ingroup robot deviated from the ingroup prototype and dissented, the derogation directed towards the robot could be interpreted as the participant psychologically distancing themselves from such deviancy to preserve positive group social identity. Alternatively, the observed BSE could reflect general expectations of robots as docile and subservient [62]. However, this explanation fails to address the differential evaluations of Psyc Bot and Eng Bot, as dissenting ingroup robots were significantly more harshly evaluated than dissenting outgroup robots. This evaluative difference based on grouping therefore adds support to the suggestion that social identity processes affected robot evaluations.

Warm robots were not preferred over cold robots. This finding contradicts previous research which demonstrated robot body language can enhance perception of robot warmth [45],[63]. However, these previous studies manipulated not only nonverbal behaviour, but other richer forms of emotional expression such as verbal expression. In this study, while posture and eye gaze differed between warm and cold robots, robot verbal behaviour and general movement was identical. The NAO robots also lacked facial expressiveness and were therefore unable to express uniquely human warmth behaviours such as smiling and head nodding [64]. Niewiadomski, Demeure, & Pelachaud [63] showed that the more modalities used by a technological agent (e.g., facial expression, prosody, gestures) the greater the agent believability and perceived warmth. Thus, the manipulation of only eye gaze and posture may have been too limited and rudimentary to elicit participant perception of robot warmth.

Alternatively, participants may have been sceptical of cues displayed by warm robots. As research suggests people scrutinise warmth behaviours to determine their legitimacy against potential deception [48], participants may have detected a mismatch between robot warmth and motive, with warm robots rated lower when they were also disagreeable. Some evidence supported this; ingroup robots were rated higher on warmth only when they were also high in competence. Thus, for robot warmth to elicit positive evaluations the robot may also need to appear proficient, so that robot motives are congruent with their ability (i.e., robots are warm in appearance and behaviour). Conversely, if robot warmth is not an important consideration for human teammates no differences in preference between warm and cold robots would be expected. This finding supports those of Hancock et al. [49] that performance factors outweigh attribute factors such as warmth. More research however is needed to investigate these alternative explanations to elucidate the influence of warmth in human-robot working groups.

This study demonstrated a BSE towards a technological agent can occur. Findings indicated deviant ingroup robots affected group social identity, and were consequently responded to (i.e., derogated) similarly to fellow human members. The importance of investigating deviance in human-robot interaction is the knowledge of which norms are salient in human-robot working groups (and are hence punished when violated). If perceptions of robots are mediated by their conformity to group norms, this may hold important implications for human-robot interaction. For instance, favourable news (e.g., promotion, task completion) could be presented by ingroup robots, and unfavourable news (e.g., task failure, incident) delivered by outgroup robots to preserve ingroup positivity. Such relationships between grouping and norm compliance could hence inform group composition and task allocation in important industries including the military, healthcare and education.

Though the present study did not find robot warmth to influence evaluations, this does not preclude warmth as a norm in human-robot working groups. Future studies could investigate human-robot interaction in contexts where robot warmth may be salient (e.g., customer service, hospitality), and hence more closely linked to group social identity. Additional research might also consider different means of measuring robot competence (beyond dissent) and how this interacts with robot warmth and teammate evaluations.

Lastly, it must be noted that a limitation of this study is that a manipulation check was not performed for a sense of belonginess or affiliation of participants to the university or their psychology discipline.
